# A New Lamellar Gold Thiolate Coordination Polymer, [Au(*m*-SPhCO_2_H)]_n_, for the Formation of Luminescent Polymer Composites

**DOI:** 10.3390/nano9101408

**Published:** 2019-10-02

**Authors:** Oleksandra Veselska, Nathalie Guillou, Gilles Ledoux, Chia-Ching Huang, Katerina Dohnalova Newell, Erik Elkaïm, Alexandra Fateeva, Aude Demessence

**Affiliations:** 1 Univ Lyon, Université Claude Bernard Lyon 1, Institut de Recherches sur la Catalyse et l’Environnement de Lyon (IRCELYON), UMR CNRS 5256, 69626 Villeurbanne, France; oleksandra.veselska@ircelyon.univ-lyon1.fr; 2 Institut Lavoisier de Versailles (ILV), UVSQ, Université Paris-Saclay, UMR CNRS 8180, 78035 Versailles, France; nathalie.guillou@uvsq.fr; 3 Univ Lyon, Université Claude Bernard Lyon 1, Institut Lumière Matière (ILM), UMR CNRS 5306, 69626 Villeurbanne, France; gilles.ledoux@univ-lyon1.fr; 4 Institute of Physics, University of Amsterdam, Science Park 904, 1098 XH Amsterdam, The Netherlands; c.huang@uva.nl (C.-C.H.); k.newell@uva.nl (K.D.N.); 5 Beamline Cristal, Synchrotron Soleil, 91192 Gif-sur-Yvette, France; erik.elkaim@synchrotron-soleil.fr; 6 Univ Lyon, Université Claude Bernard Lyon 1, Laboratoire des Multimatériaux et Interfaces (LMI), UMR CNRS 5615, 69626 Villeurbanne, France; alexandra.fateeva@univ-lyon1.fr

**Keywords:** gold thiolate, coordination polymer, lamellar structure, luminescence, polymer composite

## Abstract

The photoluminescence of gold thiolate clusters brings about many potential applications, but its origin is still elusive because of its complexity. A strategy in understanding the structure–properties relationship is to study closely related neutral gold thiolate coordination polymers (CPs). Here, a new CP is reported, [Au(*m*-SPhCO_2_H)]_n_. Its structure is lamellar with an inorganic layer made of Au–S–Au–S helical chains, similar to the [Au(*p*-SPhCO_2_H)]_n_ analog. An in-depth study of its photophysical properties revealed that it is a bright yellow phosphorescent emitter with a band centered at 615 nm and a quantum yield (QY) of 19% at room temperature and in a solid state. More importantly, a comparison to the *para*-analog, which has a weak emission, displayed a strong effect of the position of the electron withdrawing group (EWG) on the luminescent properties. In addition, [Au(*m*-SPhCO_2_H)]_n_ CPs were mixed with organic polymers to generate transparent and flexible luminescent thin films. The ability to tune the emission position with the appropriate contents makes these nontoxic polymer composites promising materials for lighting devices.

## 1. Introduction

Gold thiolate clusters, Au_n_(SR)_m_, are an intriguing family of materials that bridges the gap between molecular species and functionalized nanoparticles [1,2,3,4]. One of their attractive properties is their photoluminescence, which brings about many potential applications in areas such as chemical sensing, bioimaging, cell labeling, phototherapy, and drug delivery [5,6,7,8]. Nevertheless, the origin of their photoemission is still elusive, and the structure–properties relationship is difficult to predict. Indeed, in gold thiolate clusters, different parameters such as the gold core’s composition and geometry, the functionality of the ligands, the length and organization of the Au(I)–SR shell, their aggregation, their rigidification, and the scale effect make those systems complicated to study and rationalize in terms of the effect of each parameter [9,10,11]. The limited amount of available crystallographic structures of luminescent gold thiolate clusters precludes an understanding of the structure–properties relationship. Our strategy to attempt to rationalize the photoluminescent properties of gold thiolate clusters has been to study their analogs, neutral gold thiolate coordination polymers (CPs) [Au(SR)]_n_, which are also highly photoluminescent [12,13,14,15,16,17,18]. In crystalline CPs, all atoms are well organized on a micrometer scale, so their structure–properties relationships appear to be easier to correlate. In addition, gold thiolate CPs are the synthesis precursors of gold thiolate clusters and are consequently easier to obtain through their isolation before the addition of a reducing agent.

Even if gold thiolate CPs have been known for a long time, only four crystallographic structures have been reported so far [19]. Two are 1D CPs made of interpenetrated helical chains of Au–S–Au chains, and two are lamellar structures. The latter ones are 2D materials: in [Au(*p*-SPhCO_2_H)]_n_, the inorganic layers are made of helical gold–sulfur chains packed together through parallel aurophilic interactions [14], while in [Au(*p*-SPhCO_2_Me)]_n_, the Au–S chains have a zigzag shape and interact through perpendicular aurophilic bonds [13] (Figure S1a,b, respectively). This difference is explained by the presence of catemeric hydrogen bonds between the carboxylic acids of [Au(*p*-SPhCO_2_H)]_n_ (which implies highly bent S–Au–S angles) when such bonds are quasilinear in the ester analog. Consequently, this change in the lamellar structure induces different photoluminescent properties. [Au(*p*-SPhCO_2_Me)]_n_ exhibits a bright orange emission at 645 nm with a QY of around 70% at room temperature (RT). [Au(*p*-SPhCO_2_H)]_n_ has a double emission in blue (490 nm) and red (660 nm) at 260 K andits QY is below 1% at RT. The first emission band originates from the ligand, and the second one originates from a ligand-to-metal charge transfer (LMCT). Their intensity evolves differently with temperature and results in thermoluminochromism with good sensitivity for thermometry [14].

In order to evaluate the effect of the position of the substituent on the structure and the photoluminescent properties, *meta*-mercaptobenzoic acid (*m*-HSPhCO_2_H) was used as a ligand to isolate [Au(*m*-SPhCO_2_H)]_n_. This new gold thiolate CP also has a 2D lamellar structure and exhibits bright photoemission. In addition, its easy integration into organic polymers shows its great potential for the fabrication of flexible and transparent luminescent devices.

## 2. Materials and Methods

### 2.1. Materials

Tetrachloroauric acid trihydrate (HAuCl_4_·3H_2_O, ≥49% Au basis) and tetrahydrofuran (THF) were purchased from Alfa Aesar (Haverhill, MA, USA), and 3-mercaptobenzoic acid (*m*-HSPhCO_2_H) (>97%) was purchased from TCI (Portland, OR, USA). Ethanol and dimethylformamide (DMF) were purchased from VWR (Radnor, PA, USA). Polyvinylidene fluoride (PVDF) and poly(9-vinylcarbazole) (PVK) were purchased from Alfa Aesar and Sigma Aldrich (St. Louis, MO, USA), respectively. All reagents were used without further purification.

### 2.2. Synthesis of [Au(m-SPhCO_2_H)]_n_

A solution of HAuCl_4_·3H_2_O (100 mg, 0.25 mmol, 1 equiv.) in H_2_O (10 mL) was added to *m*-HSPhCO_2_H (178 mg, 1.15 mmol, 4.6 equiv.). The reaction was allowed to proceed for 18 h at 120 °C in a 20-mL sealed glass vial. White precipitate was obtained and washed with 40 mL of ethanol 3 times. The powder was recovered by centrifugation at 4000 rpm. The product was dried in air. The yield was 77% (68 mg); the chemical formula was C_7_H_5_AuO_2_S; the molecular weight was 350.14; and the gold content from thermogravimetric analysis (TGA) (calculated) wt% was 56.4 (56.3).

### 2.3. Synthesis of the Polymer Composites

Here, a 0.5 to 15 wt% of CPs was mixed with the organic polymer PVDF or PVK and then dispersed in a solvent, DMF (8 mL) or THF (5 mL), respectively. The total mass of the polymer composite was 400 mg. The mixture was ultrasonicated until a homogeneous emulsion was obtained. Then it was deposited onto a flat substrate (i.e., a glass slide or a Petri dish), and the solvent was evaporated at 60 °C [20,21]. 

### 2.4. Structure Determination

The structural determination of [Au(*m*-SPhCO_2_H)]_n_ was carried out from high-resolution powder X-ray diffraction data. They were collected on a CRISTAL beamline at Soleil Synchrotron (Gif-sur-Yvette, France). A monochromatic beam was extracted from the U20 undulator beam by means of a Si(111) double monochromator. Its wavelength of 0.79276 Å was refined from a LaB_6_ (NIST (National Institute of Standards and Technology) Standard Reference Material 660a) powder diagram recorded prior to the experiment. The X-ray beam was attenuated in order to limit radiation damage to the sample. A high angular resolution was obtained with (in the diffracted beam) a 21 perfect crystals Si(111) multi-analyzer. The sample was loaded in a 0.7-mm capillary (Borokapillaren, GLAS, Schönwalde, Germany) mounted on a spinner rotating at about 5 Hz to improve the particles’ statistics. Diffraction data were collected in continuous scanning mode, and a diffractogram was obtained from the precise superposition and addition of the 21-channel data.

**Figure 1:**
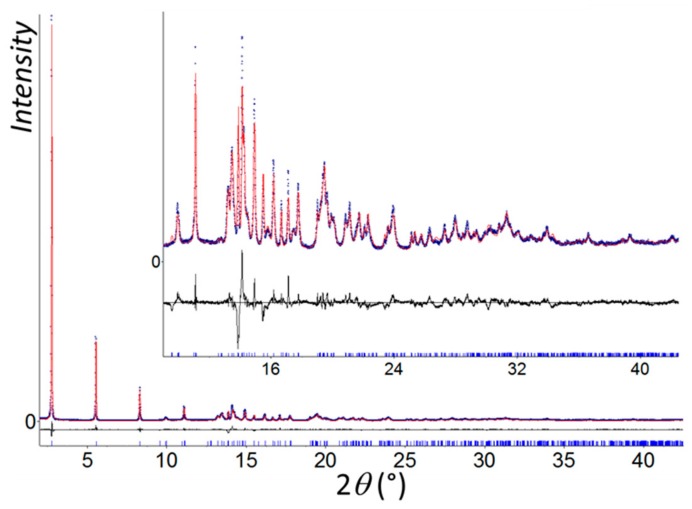
Final Rietveld plot of [Au(*m*-SPhCO_2_H)]_n_ showing observed (blue circles), calculated (red line), and difference (black line) curves. Zoomed-in high angles are shown as an inset.

All calculations of structural investigation were performed with the TOPAS program [22]. The LSI (Least Squares Iterative) indexing method converged to two possible unit cells, a monoclinic *C* cell (*a* = 6.8749(4), *b* = 6.5715(3), *c* = 16.3904(6) Å, *β* = 91.199(7)°, and *V* = 740.3(5) Å^3^ with *M*_20_ = 57) and a triclinic one (*a* = 4.7567(5), *b* = 4.7632(6), *c* = 16.3975(5) Å, *α* = 90.780(8)°, *β* = 89.123(6)°, *γ* = 92.651(4)°, and *V* = 371.05(7) Å^3^ with *M*_20_ = 44). The highest symmetry was first logically chosen to solve the structure. Nevertheless, no structural model could be initiated in the three space groups consistent with systematic extinctions *(C*2, *Cm*, and *C*2/*m*). Moreover, a careful examination of the triclinic solution showed that the unit cell parameters were very close to those of [Au(*p*-SPhCO_2_H)]_n_. The atomic coordinates of the gold and sulfur atoms of [Au(*p*-SPhCO_2_H)]_n_ were first directly used to initiate a Rietveld refinement, which did not converge. A structural investigation of [Au(*m*-SPhCO_2_H)]_n_ was then initialized in *P*1 by using the charge flipping method, which allowed for locating two gold atoms. The direct space strategy was then used to complete the structural model, and two independent organic moieties were added to the fixed gold atomic coordinates and treated as rigid bodies in the simulated annealing process. Calculations converged to *R*_B_ = 0.054 and *R*_wp_ = 0.113. This structural model was used as the starting model in the Rietveld refinement. In its final stage, this involved the following structural parameters: 6 atomic coordinates, 6 translation and 6 rotation parameters for organic moieties as well as 4 distances and 2 torsion angles in the rigid bodies, 2 thermal factors, and 1 scale factor. The final Rietveld plot (Figure 1) corresponded with a satisfactory model indicator and profile factors (Table S1). A search for higher symmetry was undertaken by using Platon software [23], and no space group change was suggested. The crystal structure quoted the depository number CCDC-1952042.

### 2.5. Characterizations

Routine PXRD: Routine powder X-ray diffraction was carried out on a Bruker D8 Advance A25 diffractometer (Karlsruhe, Germany) using Cu Kα radiation equipped with a 1-dimensional position-sensitive detector (Bruker LynxEye). X-ray scattering was recorded between 4° and 90° (2*θ*) with 0.02° steps and 0.5 s per step (28 min for the scan). A divergence slit was fixed at 0.2°, and the detector aperture was fixed to 192 channels (2.95°).

SEM: SEM images were obtained with an FEI Quanta 250 FEG (Hillsboro, OR, USA) scanning electron microscope. Samples were mounted on stainless pads and sputtered with carbon to prevent charging during observation.

FTIR: Infrared spectra were obtained from a Bruker Vector 22 FTIR spectrometer (Richmond Scientific Ltd Unit 9; Chorley, UK) with KBr pellets at room temperature and were registered from 4000 cm^−1^ to 400 cm^−1^.

TGA: Thermogravimetric analyses were performed with a TGA/DSC 1 STARe System from Mettler Toledo (Columbus, OH, USA). Around 5 mg of the sample was heated at a rate of 10 °C/min in a 70-μL alumina crucible under air (20 mL/min).

UV-VIS: A UV-VIS absorption spectrum was carried out with a LAMBDA 365 UV/VIS Spectrophotometer from Perkin Elmer (Waltham, MA, USA) in solid state at room temperature.

Photoluminescence (PL) excitation and emission spectra measurements were performed on a homemade apparatus. The sample was illuminated by an EQ99X laser-driven light source (Energetiq Technology Inc.; Woburn, MA, USA) filtered by a Jobin Yvon Gemini 180 monochromator (Paris, France). The exit slit from the monochromator was then reimaged on the sample by two MgF_2_ lenses 100 m in focal length and 2 inch in diameter. The whole apparatus was calibrated by means of a Newport 918D (Irvine, CA, USA) Low power-calibrated photodiode sensor over the range 190–1000 nm. The resolution of the system was 4 nm. The emitted light from the sample was collected by an optical fiber connected to a Jobin-Yvon TRIAX320 monochromator equipped with a cooled charge coupled device (CCD) detector (Andor Newton 970; Oxford Instruments; Abingdon, UK). At the entrance of the monochromator, different long-pass filters could be chosen in order to eliminate the excitation light. The resolution of the detection system was better than 2 nm.

Temperature control over the sample was regulated with a THMS-600 heating stage with a T95-PE temperature controller from Linkam Scientific Instruments (Epsom, UK).

Luminescence lifetime measurements: Luminescence lifetime measurements were excited with a diode-pumped 50-Hz tunable optical parametric oscillator (OPO) laser from EKSPLA (Vilnuis, Lithuania). The luminescence of the sample was collected with an optical fiber and was afterwards filtered by a long-pass filter (FEL450 from Thorlabs; Newton, NJ, USA) and the monochromator described before and fed to an R943-02 photomultiplier tube from Hamamatsu (Hamamatsu, Japan). Photon arrival times were categorized by an MCS6A multichannel scaler from Fast ComTec (Munich, Germany).

The collected data cannot be fitted by a sum of simple exponential decays. For this reason, one stretched exponential decay is often used (Equation (1)) [24]:

(1) I=a1e−(xt1)β1+a2e−xt2+a3e−xt3,

with $a_{i}$ and $t_{i}$ being the amplitude and lifetime of a given component *i*, and $\beta_{1}=\left[0,\;1\right]$.

The distribution of a lifetime with a *β* factor is generally associated with the possibility of energy transfer toward a distribution of nonradiative centers through dipole–dipole/quadrupole–quadrupole, etc., interactions. Its value is defined by the types of interactions and the dimensionality of the system.

The average lifetime of the stretched exponential decay $\langle \tau_{1}\rangle$ is calculated through Equation (2). The procedure used for the fitting is described in Reference [25]:

(2) $$\langle \tau_{1}\rangle =\tau_{1}\cdot \frac{1}{\beta_{1}}\cdot \Gamma \left(\frac{1}{\beta_{1}}\right).$$

Determination of the QY with a standard integrating sphere (IS) (shown schematically in Figure S2a): To illuminate the sample, a stabilized Xenon lamp (Hamamatsu, L2273) coupled to a double-grating monochromator (Solar, MSA130; Minsk, Belarus) was used. The excitation beam was split using a spectrally broad bifurcated fiber (Ocean Optics, BIF600-UV-VIS; Duiven, The Netehrlands), where one part was used to monitor the fluctuations of the excitation intensity using a power meter (Ophir Photonics, PD300-UV; Jerusalem, Israel) and the other part was used to excite the sample. A collimator lens was used to reduce the spot size at the sample position to enable direct excitation of the sample (*F* = 1). The powder samples were placed in ethanol solution in a quartz cuvette and suspended using an aluminum holder in the center of the IS (internal walls made of Spectralon^®^ (PTFE), Newport, 70672) (with a diameter of 10 cm). The use of this type of holder has been verified using ray-tracing simulations [26]. Light was detected using a second spectrally broad optical fiber (Ocean Optics, QP1000-2-VIS-BX) coupled to a spectrometer (Solar, M266) equipped with a CCD (Hamamatsu, S7031-1108S). The sample (powder) did not dissolve completely in the solvent (ethanol), so the sample was shaken before the measurement and then measured. This was repeated several times to ensure reproducibility of the results. All measurements were corrected for the spectral response of the detection system, which we determined by illuminating the IS via the excitation port with a tungsten halogen calibration lamp (standard of spectral irradiance, Oriel, 63358; Irvine, CA, USA) for the visible range and a deuterium lamp (Oriel, 63945) for the UV range (<400 nm). The measured calibration spectrum was corrected for the spectrometer’s stray-light contribution. Reabsorption effects were corrected for using the procedure described by Ahn et al. [27] by comparing the measured PL spectrum to that of a low-concentration sample for which reabsorption was negligible. Error estimates were obtained following Chung et al. [28]. QY was evaluated using Equation (3), where $N_{em}$ and $N_{abs}$ are numbers of emitted and absorbed photons, and $N_{S}$ and $N_{Ref}$ are numbers of photons transmitted through the sample (sample in solvent and cuvette) and reference (just cuvette with solvent) (star denotes emission spectral range, and no star means excitation spectral range). $I_{S}$ and $I_{Ref}$ are emission intensities detected within the sample and reference (Figure S3b), and *C* is a correction factor for the spectral response of the detection system:

(3) $$QY=\frac{N_{em}}{N_{abs}}=\frac{N_{S}^{*}-N_{Ref}^{*}}{N_{Ref}-N_{S}}=\frac{\int^{em}\left[I_{S}\left(\lambda \right)-I_{Ref}\left(\lambda \right)\right]C\left(\lambda \right)d\lambda }{\int^{exc}\left[I_{Ref}\left(\lambda \right)-I_{S}\left(\lambda \right)\right]C\left(\lambda \right)d\lambda }.$$

## 3. Results and Discussion

### 3.1. Synthesis and Characterization

Pure and highly crystalline powder of [Au(*m*-SPhCO_2_H)]_n_ CP was synthesized under hydrothermal conditions. The procedure was similar to the one used for the *para*-substituted analog, [Au(*p*-SPhCO_2_H)]_n_ [14]. The PXRD pattern (Figure 1) showed predominant (00*l*) reflections underlining the lamellar structure of this material, with an interlamellar distance of 16.3 Å.

The morphology of the crystallites was thin pellets typical of lamellar compounds, as shown by SEM images (Figure S3). In the FTIR spectroscopy, the CO antisymmetric vibration band of the carboxylic acid was observed at 1683 cm^−1^, the same position as for the free ligand, which showed that the carboxylic acids were not coordinated with the metal atoms. In addition, the broad ν(OH) bands between 2560 and 3065 cm^−1^ were consistent with the presence of hydrogen bonds between the carboxylic acid functions (Figure S4). The thermogravimetric analyses confirmed the purity of the compound with an expected 1:1 metal/organic content. This CP showed good thermal stability up to 300 °C under air (Figure S5), which was comparable to [Au(*p*-SPhCO_2_H)]_n_ (320 °C) [14].

The synthesis did not result in obtaining single crystals, and thus a structural determination from the high-resolution PXRD data was carried out.

### 3.2. Structure

**Figure 2:**
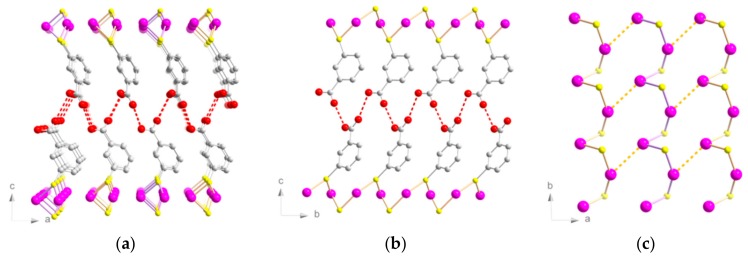
Structure representations of [Au(*m*-SPhCO_2_H)]_n_. Views of (**a**) (*ac*) and (**b**) (*bc*) planes. (**c**) View of the Au–S chains on the (*ab*) plane. Pink, Au; yellow, S; red, O; grey, C. Hydrogen atoms are omitted for clarity. Red and orange dotted lines represent the hydrogen bonds and interchain aurophilic interactions, respectively.

The lamellar structure was triclinic (*P*1 space group) and was similar to the previously discussed [Au(*p*-SPhCO_2_H)]_n_ [4c]. It consisted of planes made of 1D helices of Au(I) atoms bridged by *µ*_2_-S atoms (Figure 2). The Au–S distances were between 2.28(2) and 2.36(2) Å (Table S2). The Au–S–Au angles were 85.8(4)° and 90.5(4)°. The S–Au–S angles were 88.2(4)° and 151.1(5)°, far from the linear angle usually observed in dicoordinated gold atoms.

In the lamellar [Au(*p*-SPhCO_2_Me)]_n_ CP without hydrogen bonds, the S–Au–S angles were close to linear (177.5(1)°) [13]. Thus, the formation of such unusual S–Au–S angles in [Au(*m*-SPhCO_2_H)]_n_ was driven by the formation of both catemeric hydrogen bonds and aurophilic interactions, similarly to [Au(*p*-SPhCO_2_H)]_n_ [14]. The interchain Au–Au distances were slightly longer than in [Au(*p*-SPhCO_2_H)]_n_ (3.38(2) and 3.61(2) Å for the *meta* vs 3.36(1) and 3.42(1) Å for the *para*), while the intrachain Au–Au distances were shorter here than in the *para*-substituted counterpart (3.21(2) and 3.28(2) Å for the *meta* vs 3.59(1) and 3.73(1) Å for the *para*).

### 3.3. Photophysical Properties

The UV light excitation of the [Au(*m*-SPhCO_2_H)]_n_ resulted in a very bright yellowish-orange emission at room temperature.

The maximum absorption of [Au(*m*-SPhCO_2_H)]_n_ was at 320 nm (the optical band gap was 2.9 eV) (Figure S6). The absorption edge was red-shifted in comparison to the free ligand. The high-energy absorption was assigned to a π–π* transition of the phenyl group of the ligand [29].

**Figure 3:**
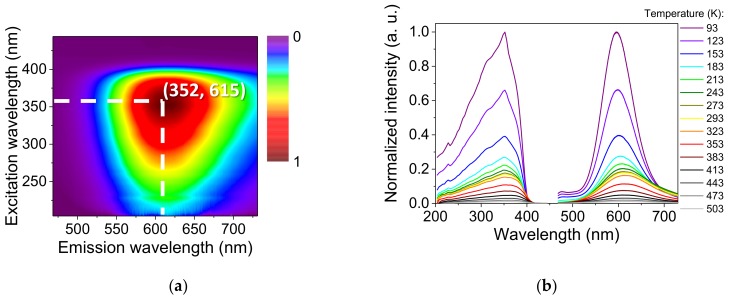
Luminescent properties of [Au(*m*-SPhCO_2_H)]_n_: (**a**) 2D map of the emission and excitation spectra conducted in a solid state at room temperature; (**b**) emission and excitation spectra (λ_exc_ = 352 nm, λ_em_ = 596 nm) in a solid state at varying temperatures.

At room temperature, the emission and excitation maxima were positioned at 615 and 352 nm, respectively (Figure 3a). Upon a temperature decrease, the emission intensity increased with a soft refinement of the width and a shift of the emission maximum to 595 nm at 93 K (Figure 3b). Upon a temperature increase, no important shift of emission relative to RT was observed. Meanwhile, the excitation maximum slightly shifted to 368 nm at 503 K (Figure S7).

Additionally, there was a low-intensity emission shoulder at 475 nm that appeared below 213 K. The free ligand exhibited a large band of emission centered at 507 nm under the same excitation wavelength. Thus, this high-energy emission could be assigned to a metal-perturbed intraligand (IL) transition. Similar behavior has been observed before in Au(I) phosphane complexes containing a 4-nitrophenylthiolate ligand [30] and dinuclear neutral thiolate Au(I) complexes with phenylene spacers [31].

The luminescence lifetime decay in solid state at room temperature was fitted with a triexponential curve with components of 0.31 µs (88%), 0.14 ms (10%), and 2.3 ms (2%) (Figures S8 and S9, Table S3). The lifetime decay study at various temperatures showed a shortening of the lifetime of the major components upon an increase of the temperature: at 93 K the lifetime was 0.87 µs, and at 503 K it dropped to 0.04 µs (Figure S10). Its contribution at low temperatures stayed at the level of 86%, and it grew up to 94% at high temperatures. Two smaller components experienced minor changes in lifetime in the studied temperature range. The contribution of both decreased upon temperature increase. The lifetime shortening could be attributed to thermal quenching. This results from thermal activation of the nonradiative decay pathways [32,33].

The long lifetime in the microsecond range and the large Stokes shift of 12,150 cm^−1^ (RT) were both characteristic of a phosphorescence process. The QY was 18.9% ± 0.2% in solid state at RT.

The close emission and excitation energies, the lifetime decay behavior, the low-intensity emission shoulder, and the high QY in solid state made the photophysical properties of [Au(*m*-SPhCO_2_H)]_n_ very similar to [Au(*p*-SPhCO_2_Me)]_n_ [13]. The overall luminescence process of [Au(*p*-SPhCO_2_Me)]_n_ was ascribed to a ligand–metal-to–ligand charge transfer transition (LMLCT: AuS → PhCO_2_Me) based on Density Functional Theory calculations. Strong similarities in the properties of the two CPs suggested the same luminescence origin for [Au(*m*-SPhCO_2_H)]_n_ as the one proposed for [Au(*p*-SPhCO_2_Me)]_n_–LMLCT. This supports the hypothesis that the presence of an electron withdrawing group (EWG) precludes the possibility of LM(M)CT transitions, which is often ascribed to Au(I) compounds.

It is interesting to note that despite the fact that [Au(*m*-SPhCO_2_H)]_n_ was structurally related to [Au(*p*-SPhCO_2_H)]_n_, they had different luminescent properties. Moreover, as was shown above, the properties were closer to the ones of [Au(*p*-SPhCO_2_Me)]_n_, which differs structurally and possesses disparate Au–S arrangements (helices vs zigzags). The aurophilic interactions and substituents on the organic ligand are often the main parameters that can help to trace the origin of the photophysical properties [34]. The presence of aurophilic interactions is known to influence photoluminescent processes: their length, dimensionality (dimer or chain), and intra/interconnectivity play an intricate role in the wavelength and intensity of the emission [34,35].

Considering the Au–Au distances (Table S2), the intrachain Au–Au distances (two gold atoms bridged by one sulfur atom) were shorter in the *meta*-compound than in its *para*-analog: 3.21(2) and 3.28(2) Å versus 3.59(1) and 3.73(1) Å. Nevertheless, the interchain Au–Au distances generating chains in the case of [Au(*p*-SPhCO_2_H)]_n_ were slightly shorter (3.36(1) and 3.42(1) Å) than the ones of [Au(*m*-SPhCO_2_H)]_n_, for which there was one aurophilic interaction (3.38(2) Å) forming dimers, the second one being too long (3.61(2) Å) to be considered. In the case of [Au(*p*-SPhCO_2_Me)]_n_, the intrachain Au–Au distance was rather long (3.51(1) Å), and the interchain one was short (3.20(1) Å).

In the structural and photophysical data obtained for the three 2D gold thiolate CPs, an interesting trend could be seen. It appears that the two compounds with the shortest Au–Au distances, [Au(*m*-SPhCO_2_H)]_n_ (3.20(2) Å, intrachain) and [Au(*p*-SPhCO_2_Me)]_n_ (3.20(1) Å, interchain), exhibited higher energy emissions at 615 and 645 nm and higher QYs of 19% and 70%, respectively, than did [Au(*p*-SPhCO_2_H)]_n_, which had an Au–Au distance of 3.36(1) Å (interchain) and showed an emission at 665 nm with a QY of less than 1% at RT. Thus, an increase of the shortest Au–Au distances was followed by a decrease of the QY. In the literature, the effect of aurophilic interactions on the quantum yield is still under debate. Shorter aurophilic interactions are often associated with better photophysical properties [36]. However, sometimes the opposite is reported [37].

The difference in photophysical properties might also be explained by the change in substituent position on the phenyl ring and the stronger effect of the carboxylic acid EWG in the *meta*-position compared to the *para*-analog. EWG groups on the ligand stabilize the sulfur highest occupied molecular orbital (HOMO), making the ligand more difficult to oxidize, inducing a shift of the emission to higher energies [34,38]. Finally, it appears that the position change of an EWG for similar structures could induce different aurophilic interactions and a significant increase of the QY from ~1% for [Au(*p*-SPhCO_2_H)]_n_ to 19% for [Au(*m*-SPhCO_2_H)]_n_.

### 3.4. Polymer Composite Films

In order to integrate this bright luminescent CP in devices for lighting applications, films were elaborated [20,39]. Thus, the pellet-like crystals of [Au(*m*-SPhCO_2_H)]_n_ were used for the fabrication of homogeneous thin films that exhibited a bright luminescent response. Two organic polymers were chosen: polyvinylidene difluoride (PVDF), which is nonluminescent, and poly(9-vinylcarbazole) (PVK), which is blue-emissive.

**Figure 4:**
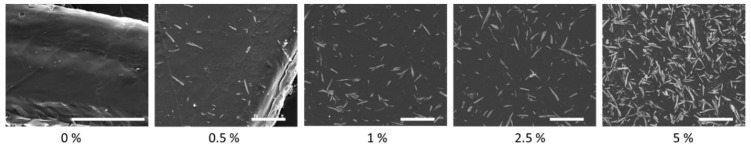
SEM images of the [Au(*m*-SPhCO_2_H)]_n_@ PVDF films with the different wt% loadings of the CP. The scale bar is 50 µm.

**Figure 5:**
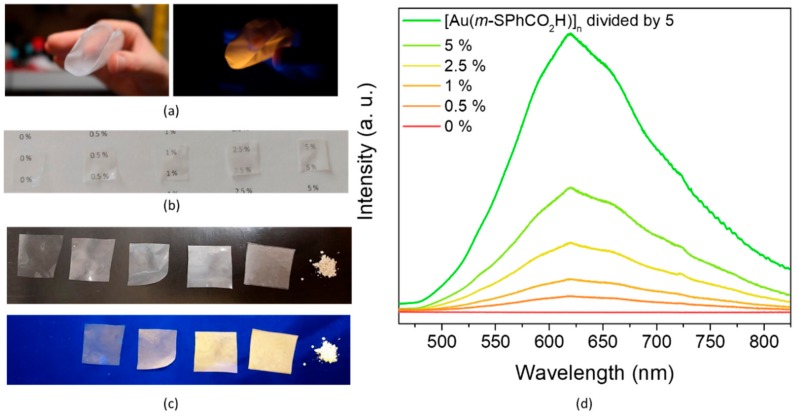
Photographs of (**a**) 2.5 wt% [Au(*m*-SPhCO_2_H)]_n_@PVDF film; and films with 0 wt%, 0.5 wt%, 1 wt%, 2.5 wt%, and 5 wt% [Au(*m*-SPhCO_2_H)]_n_@PVDF showing their (**b**) transparence and (**c**) luminescence. (**d**) Emission spectra (λ_ex_ = 380 nm) of these polymer composite films insolid state at room temperature.

PVDF films containing 0.5 wt%, 1 wt%, 2.5 wt%, and 5 wt% [Au(*m*-SPhCO_2_H)]_n_ were prepared. The PXRD showed that after film preparation, the CPs remained crystalline (Figure S11). SEM images indicated good dispersion of the CP crystallites in the PVDF following an increase in their density with an increase in the loading (Figure 4). In addition, the high QY of [Au(*m*-SPhCO_2_H)]_n_ allowed for the use of a small quantity of CPs, 0.5 wt%, when obtaining transparent, flexible, and luminescent films (Figure 5a–c). The intensity of the emission increased gradually with the CP loading (Figure 5d).

**Figure 6:**
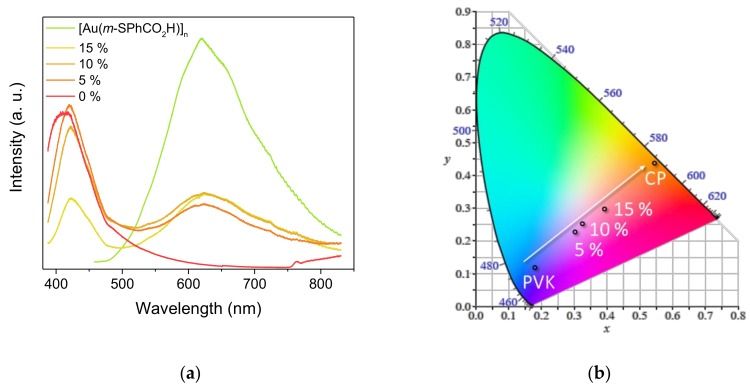
(**a**) Emission spectra of [Au(*m*-SPhCO_2_H)]_n_@PVK films at variable CP loadings; (**b**) CIE 1931 chromaticity diagram showing the luminescence color change of the [Au(*m*-SPhCO_2_H)]_n_@PVK films with variable CP loadings. Data were registered under an excitation of 370 nm in a solid state at room temperature.

An analogous approach was used for the synthesis of PVK films containing [Au(*m*-SPhCO_2_H)]_n_ (5 wt%, 10 wt%, and 15 wt% of loading). The PXRD patterns showed that the crystallinity of the CP was maintained in the polymer composites (Figure S12). PVK is a blue-emissive polymer. Thus, the mixture of PVK with yellowish-orange-emitting CP as [Au(*m*-SPhCO_2_H)]_n_ led to dual emission at 420 and 620 nm (Figure 6a). This allowed for fine-tuning the emission color from blue to orange by adjusting the quantity of the CP loading. This variable loading study specifically showed that flexible films with an emission close to white light could be obtained as shown in CIE 1931 (Comission Internationale de l’Eclairage) chromaticity diagram (Figure 6b). Given the very high interest in the development of white-light phosphors [40], formulating such composite CP/organic polymer films appears to be a valuable method for tuning the emission wavelength and generating nontoxic materials with white-light emission.

## 4. Conclusions

Here, we report a new gold thiolate coordination polymer, [Au(*m*-SPhCO_2_H)]_n_. Its structure is lamellar, and its photophysical properties show a bright yellow emission up to 500 K with a QY of 19% in solid state and at room temperature. While its structure is similar to the analog [Au(*p*-SPhCO_2_H)]_n_, its luminescence is much more intense, displaying the effect of shorter Au–Au distances and the position of the EWG on the phenyl ring of the ligand. Related to the gold thiolate clusters, this shows that a subtle change in the structure can lead to tremendous change in the photophysical properties. Consequently, further studies on these basic CPs should be carried out to understand in-depth the photoemission of the more complicated clusters. In addition, this gold thiolate CP can be easily used along with organic polymers to formulate composite transparent, flexible, and luminescent films. In these latter materials, the emission color can be tuned by adjusting the CP loading. These abilities and their nontoxicity make gold thiolate CPs a good alternative to quantum dots for lighting devices.

## Supplementary Materials

The following are available online at https://www.mdpi.com/2079-4991/9/10/1408/s1: Figure S1 Structure representations of (a) [Au(*p*-SPhCO_2_H)]_n_ [1] and (b) [Au(*p*-SPhCO_2_Me)]_n_ [2]. Pink, Au; yellow, S; red, O; grey, C. Hydrogen atoms are omitted for clarity. Red and purple dotted lines represent the hydrogen bonds and the aurophilic interactions, respectively. Figure S2 A scheme of a used standard integrating sphere setup was used (a) and a graphical representation of emission intensities detected with sample and reference (b). Figure S3 SEM image of [Au(m-SPhCO_2_H)]n. Figure S4 FT-IR spectra of the free ligand (black) and [Au(m-SPhCO_2_H)]n (red). Figure S5 The TGA conducted under air at 10 °C/min of [Au(m-SPhCO_2_H)]n. Figure S6 UV-vis absorption spectra of [Au(m-SPhCO_2_H)]n (red) and the free ligand (black) conducted in solid state at the room temperature. Figure S7 Normalized emission-excitation spectra (λexc = 352 nm, λem = 596 nm) of [Au(m-SPhCO_2_H)]n conducted in solid state at variable temperatures. Figure S8 Temperature-dependent luminescence decay curves (λexc = 352 nm, λem = 610 nm) of [Au(m-SPhCO_2_H)]n conducted in solid state at variable temperatures. Figure S9 Examples of lifetime decay fit of [Au(m-SPhCO_2_H)]n at 93 K (a), 293 K (b) and 503 K (c). Figure S10 Temperature-dependent luminescence decays of [Au(m-SPhCO_2_H)]n: values of the lifetime (a) and contributions (b) of different components at various temperatures. Figure S11 PXRD patterns of x %[Au(m-SPhCO_2_H)]n@PVDF films. Figure S12 PXRD patterns of x %[Au(m-SPhCO_2_H)]n@PVK films. Table S1 Crystallographic data and Rietveld refinement parameters for [Au(m-SPhCO_2_H)]n. Table S2 Comparison of the main distances and angles in [Au(m-SPhCO_2_H)]n and other lamellar [Au(SR)]n CPs, [Au(p-SPhCO_2_H)]n [1] and [Au(p-SPhCO_2_Me)]n [2]. Table S3 Luminescence lifetimes (τi), their pre-exponential factors (ai), parameters β1, average lifetimes (<τi>) and contributions of [Au(m-SPhCO_2_H)]n at 93 to503 K temperature range.
