# Supplementary material for: A New Lamellar Gold Thiolate Coordination Polymer, [Au(m-SPhCO2H)]n, for the Formation of Luminescent Polymer Composites
Source: Nanomaterials (Basel). 2019 Oct 2;9(10):1408. doi: 10.3390/nano9101408 (PMC6835532; doi:10.3390/nano9101408)
Supplement: Supplementary file 1 [file nanomaterials-09-01408-s001.pdf]

Electronic Supporting Information for:

# A New Lamellar Gold Thiolate Coordination Polymer, [Au($m$-SPhCO$_2$H)]$_n$, for the Formation of Luminescent Polymer Composites

**Oleksandra Veselska [1], Nathalie Guillou [2], Gilles Ledoux [3], Chia-Ching Huang [4], Katerina Dohnalova Newell [4], Erik Elkaïm [5], Alexandra Fateeva [6] and Aude Demessence [1,*]**

1. Institut de Recherches sur la Catalyse et l'Environnement de Lyon (IRCELYON), Université Claude Bernard Lyon 1, UMR CNRS 5256, 69626 Villeurbanne, France; oleksandra.veselska@ircelyon.univ-lyon1.fr
2. Institut Lavoisier de Versailles (ILV), UVSQ, Université Paris-Saclay, UMR CNRS 8180, 78035 Versailles, France; nathalie.guillou@uvsq.fr
3. Institut Lumière Matière (ILM), Université Claude Bernard Lyon 1, UMR CNRS 5306, 69626 Villeurbanne, France; gilles.ledoux@univ-lyon1.fr
4. Institute of Physics, University of Amsterdam, Science Park 904, 1098 XH Amsterdam, The Netherlands; c.huang@uva.nl (C.-C.H.); k.newell@uva.nl (K.D.N.)
5. Beamline Cristal, Synchrotron Soleil, 91192 Gif-sur-Yvette, France; erik.elkaim@synchrotron-soleil.fr
6. Laboratoire des Multimatériaux et Interfaces (LMI), Université Claude Bernard Lyon 1, UMR CNRS 5615, 69626 Villeurbanne, France; alexandra.fateeva@univ-lyon1.fr

* Correspondence: aude.demessence@ircelyon.univ-lyon1.fr

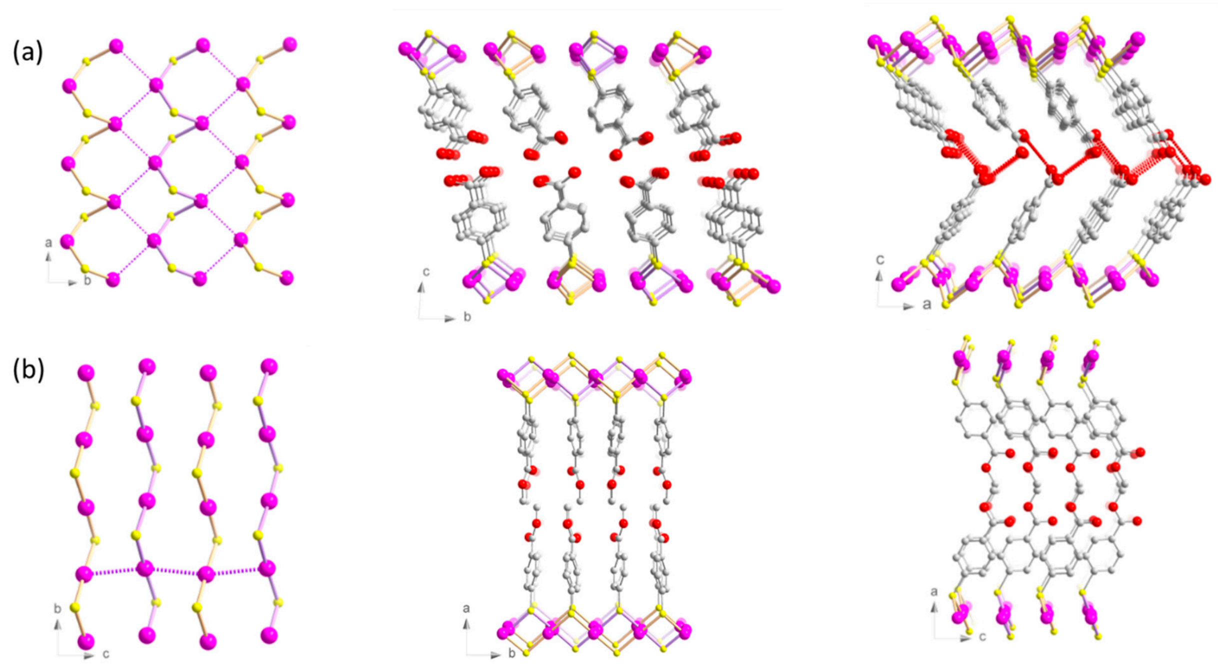


**Figure 1.** Structure representations of (a) [Au($p$-SPhCO$_2$H)]$_n$ [1] and (b) [Au($p$-SPhCO$_2$Me)]$_n$ [2]. Pink, Au; yellow, S; red, O; grey, C. Hydrogen atoms are omitted for clarity. Red and purple dotted lines represent the hydrogen bonds and the aurophilic interactions, respectively.

**Table S1 Crystallographic data and Rietveld refinement parameters** for [Au($m$-SPhCO$_2$H)]$_n$.

| **Empirical formula** | **$C_7H_5Au_1O_2S_1$** |
|---|---|
| $M_r$ | 350.15 |
| Crystal system | Triclinic |

| | |
|---|---|
| Space group | $P1$ |
| $a$ (Å) | 4.7567(5) |
| $b$ (Å) | 4.7632(6) |
| $c$ (Å) | 16.3975(5) |
| α (°) | 90.780(8) |
| $\beta$ (°) | 89.123(6) |
| $\gamma$ (°) | 92.651(4) |
| $V$ (Å$^3$) | 371.05(6) |
| Z | 2 |
| $\lambda$ (Å) | 0.79276 |
| Number of reflections | 618 |
| Number of fitted structural parameters | 27 |
| $R_p$, $R_{wp}$ | 0.061, 0.090 |
| $R_{Bragg}$, GoF | 0.037, 1.68 |

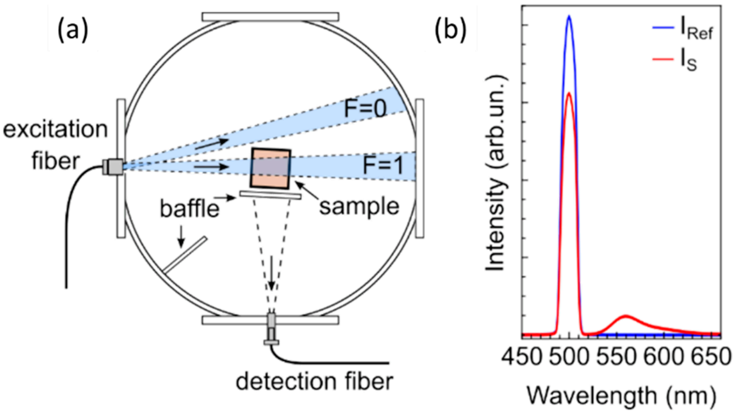


**Figure 2.** A scheme of a used standard integrating sphere setup was used (a) and a graphical representation of emission intensities detected with sample and reference (b).

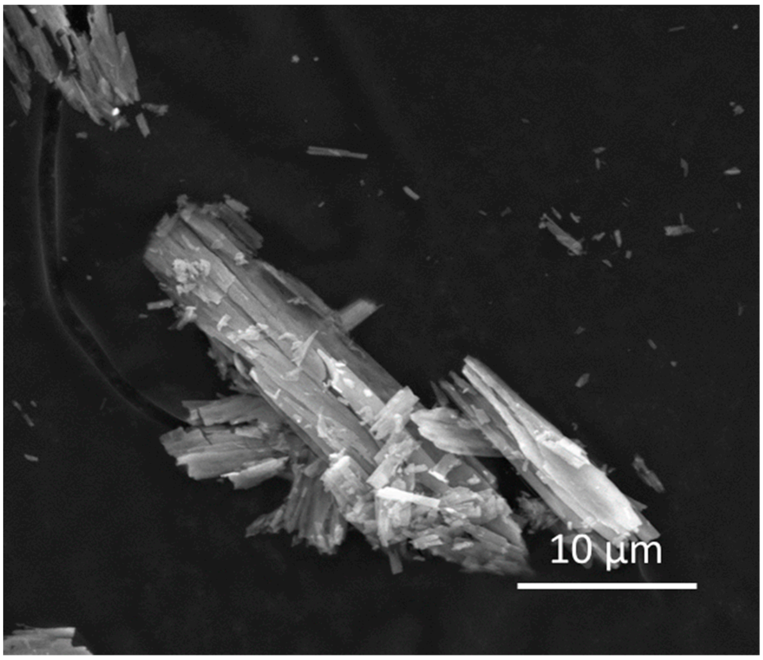


**Figure 3.** SEM image of [Au(*m*-SPhCO$_2$H)]$_n$.

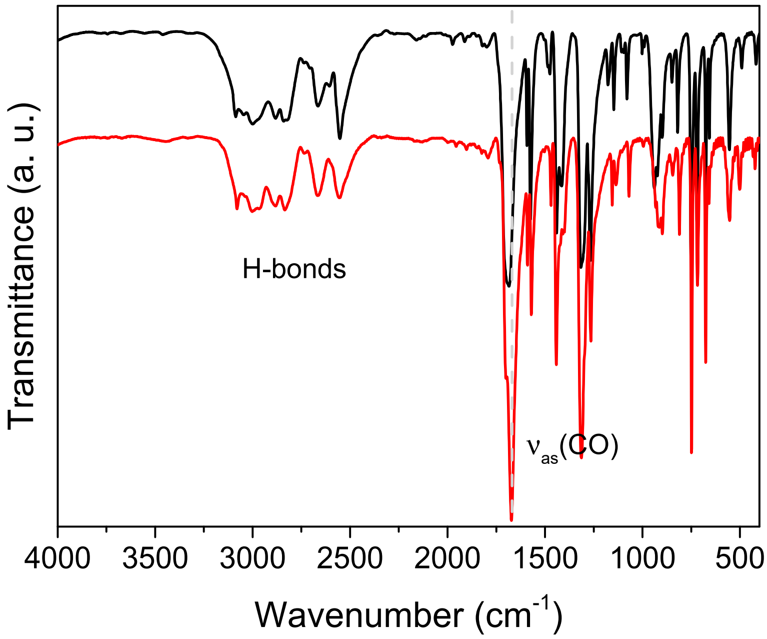


***Figure 4.*** FT-IR spectra of the free ligand (black) and [Au(m-SPhCO$_2$H)]$_n$ (red).

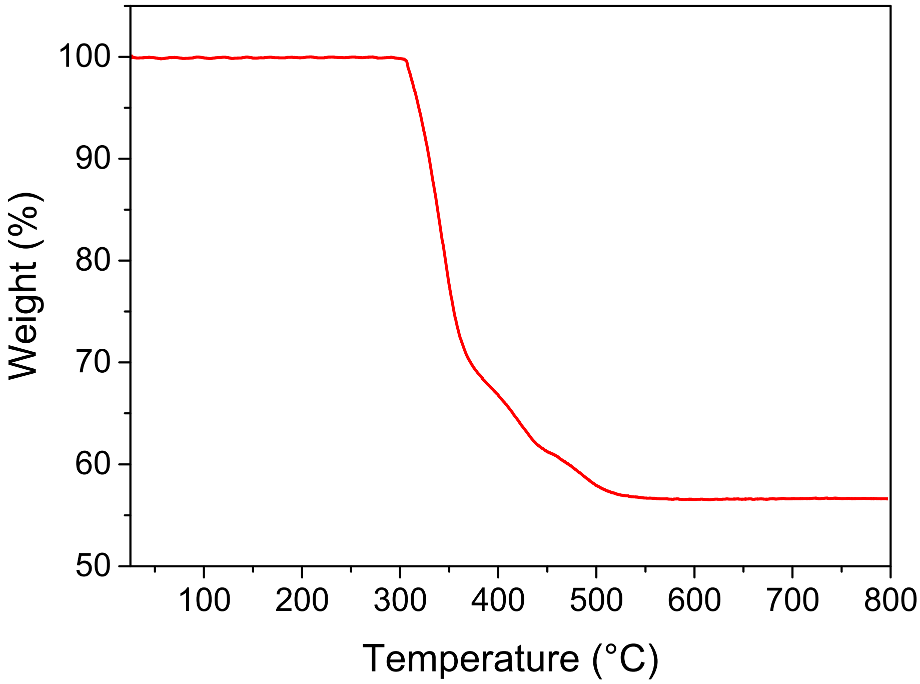


**Figure 5.** The TGA conducted under air at 10°C/min of [Au(*m*-SPhCO$_2$H)]$_n$.

**Table 2.** Comparison of the main distances and angles in [Au(*m*-SPhCO$_2$H)]$_n$ and other lamellar [Au(SR)]$_n$ CPs, [Au(*p*-SPhCO$_2$H)]$_n$ [1] and [Au(*p*-SPhCO$_2$Me)]$_n$ [2].

| Compound | Au-S Å | Au-Au Å | S-Au-S ° | Au-S-Au ° | O1-O2 Å | π-π Å | C-H… π Å |
|---|---|---|---|---|---|---|---|
| **[Au(*m*-SPhCO$_2$H)]$_n$** | 2.28(2)<br>2.35(2)<br>2.36(2) | 3.21(2)†<br>3.28(2)†<br>3.38(2)δ<br>3.61(2)δ | 88.2(4)<br>151.1(5) | 85.8(4)<br>90.5(4) | 2.714(1)<br>3.039(1) | 4.757(1) | |
| **[Au(*p*-SPhCO$_2$H)]$_n$** | 2.27(1)<br>2.34(1)<br>2.37(1) | 3.36(1)δ<br>3.42(1)δ<br>3.59(1)†<br>3.73(1)† | 77.5(3)<br>124.0(4) | 99.4(2)<br>110.4(2) | 2.903(1)<br>3.011(1) | 4.518(1) | |
| **[Au(*p*-SPhCO$_2$Me)]$_n$** | 2.31(1) | 3.20(1)δ<br>3.51(1)† | 177.5(1) | 97.1(1) | - | - | 3.233(1)<br>4.200(1) |

† Au-Au intrachain (bridged by sulfur atoms).

δ Au-Au interchain.

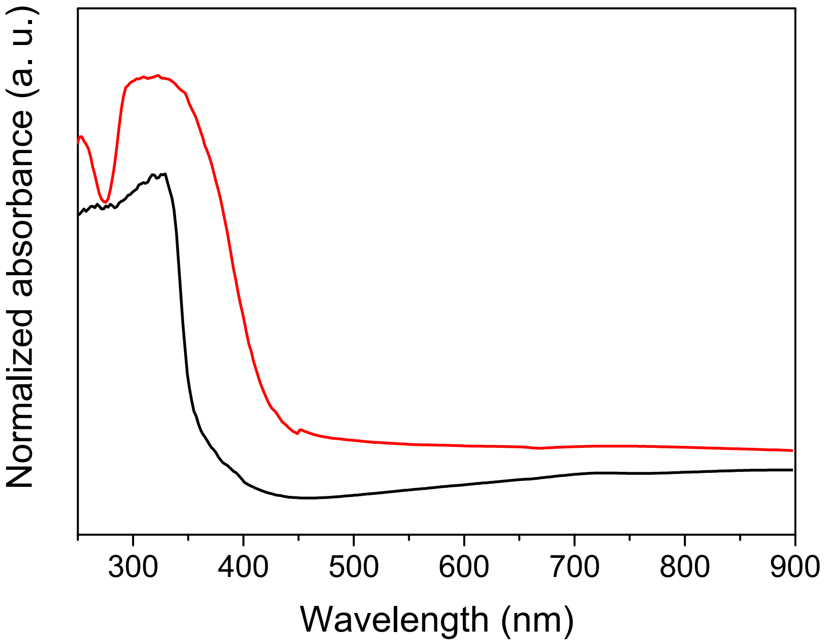


**Figure 6.** UV-vis absorption spectra of [Au(*m*-SPhCO$_2$H)]$_n$ (red) and the free ligand (black) conducted in solid state at the room temperature.

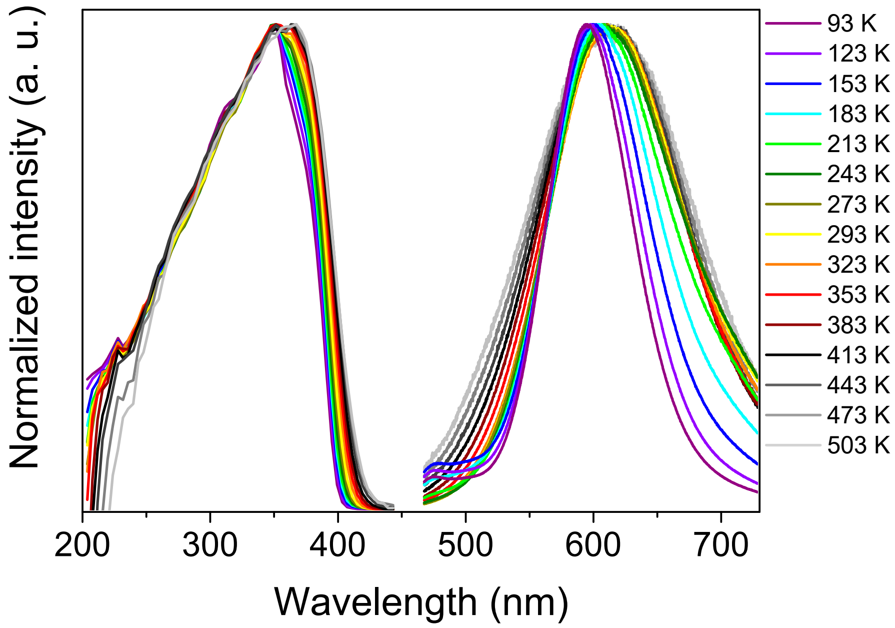


**Figure 7.** Normalized emission-excitation spectra ($\lambda_{exc}$ = 352 nm, $\lambda_{em}$ = 596 nm) of [Au(*m*-SPhCO$_2$H)]$_n$ conducted in solid state at variable temperatures.

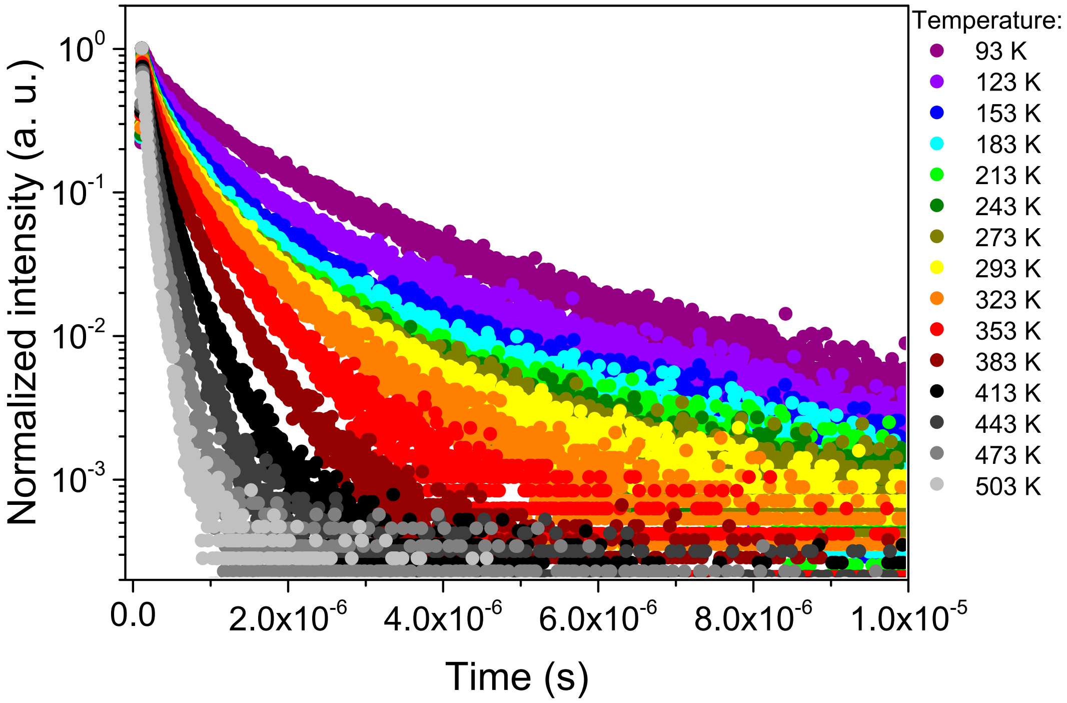


**Figure 8.** Temperature-dependent luminescence decay curves ($\lambda_{exc}$ = 352 nm, $\lambda_{em}$ = 610 nm) of [Au(*m*-SPhCO$_2$H)]$_n$ conducted in solid state at variable temperatures.

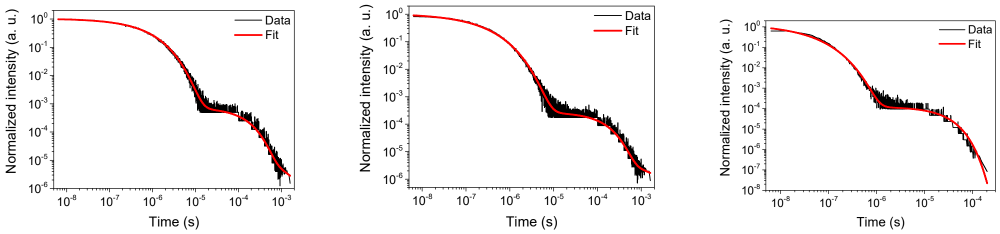


(a) (b) (c)

**Figure 9.** Examples of lifetime decay fit of [Au(*m*-SPhCO$_2$H)]$_n$ at 93 K (a), 293 K (b) and 503 K (c).

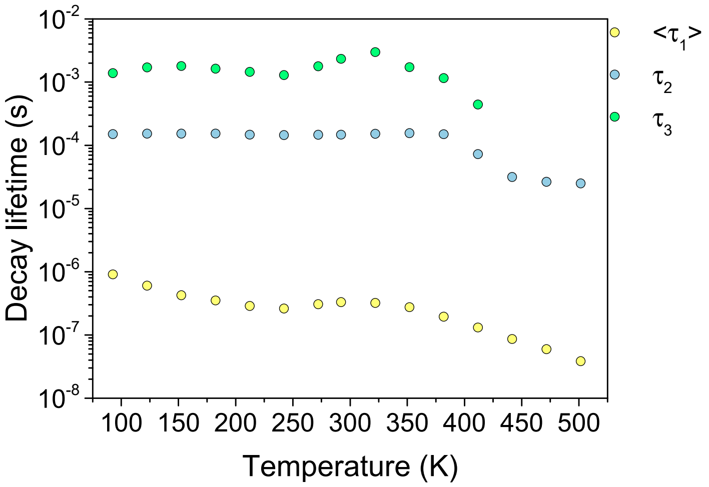


**(a).**

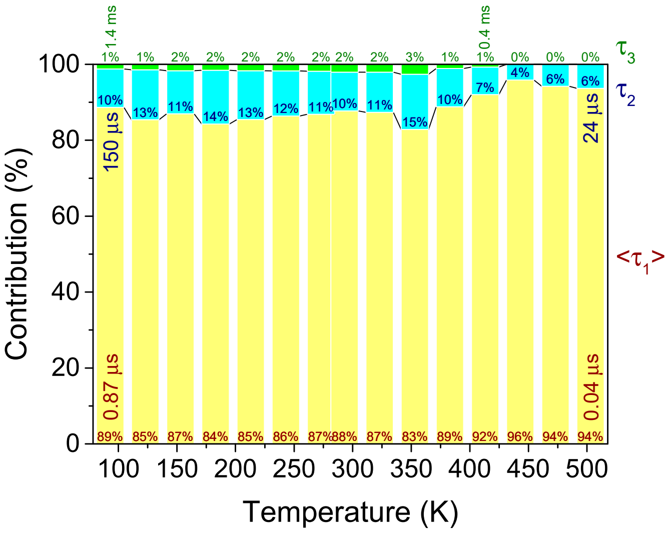


(b)

**Figure 10.** Temperature-dependent luminescence decays of [Au($m$-SPhCO$_2$H)]$_n$: values of the lifetime (a) and contributions (b) of different components at various temperatures.

**Table S2 Luminescence lifetimes ($\tau_i$), their pre-exponential factors ($a_i$), parameters $\beta_1$, average lifetimes ($\langle\tau_i\rangle$) and contributions of [Au(*m*-SPhCO$_2$H)]$_n$ at 93 t**o503 K temperature range.

| Temperature, K | $a_1 \pm \sigma a_1$ | $\tau_1 \pm \sigma\tau_1$, s | $\beta_1 \pm \sigma\beta_1$ | $\langle\tau_1\rangle \pm \sigma\langle\tau_1\rangle$, s | Contribution of $\tau_1$, % | $a_2 \pm \sigma a_2$ | $\tau_2 \pm \sigma\tau_2$, s | Contribution of $\tau_2$, % | $a_3 \pm \sigma a_3$ | $\tau_3 \pm \sigma\tau_3$, s | Contribution of $\tau_3$ % | $R^2$ |
|---|---|---|---|---|---|---|---|---|---|---|---|---|
| **93** | 1.0281 ± 0.0058 | 6.35E-07 ± 6.96E-09 | 0.649 ± 0.003 | 8.69E-07 ± 1.01E-08 | 89 | 6.84E-04 ± 4.39E-06 | 1.47E-04 ± 8.62E-07 | 10 | 8.93E-06 ± 5.37E-07 | 1.37E-03 ± 8.70E-05 | 1 | 0.99775 |
| **123** | 1.0663 ± 0.0070 | 3.67E-07 ± 4.80E-09 | 0.583 ± 0.002 | 5.74E-07 ± 7.92E-09 | 85 | 6.20E-04 ± 3.78E-06 | 1.49E-04 8.06E-07 | 13 | 5.95E-06 ± 3.90E-07 | 1.69E-03 ± 1.41E-04 | 1 | 0.99772 |
| **153** | 1.1449 ± 0.0093 | 2.26E-07 ± 3.65E-09 | 0.533 ± 0.002 | 4.05E-07 ± 6.86E-09 | 87 | 3.96E-04 ± 2.65E-06 | 1.49E-04 ± 9.46E-07 | 11 | 4.91E-06 ± 3.22E-07 | 1.77E-03 ± 1.57E-04 | 2 | 0.99741 |
| **183** | 1.1843 ± 0.0102 | 1.77E-07 ± 3.04E-09 | 0.516 ± 0.002 | 3.34E-07 ± 6.03E-09 | 84 | 4.32E-04 ± 2.72E-06 | 1.50E-04 ± 8.72E-07 | 14 | 4.42E-06 ± 3.10E-07 | 1.61E-03 ± 1.38E-04 | 2 | 0.99746 |
| **213** | 1.3044 ± 0.0128 | 1.33E-07 ± 2.59E-09 | 0.492 ± 0.002 | 2.74E-07 ± 5.63E-09 | 85 | 3.63E-04 ± 2.45E-06 | 1.44E-04 ± 9.28E-07 | 13 | 4.80E-06 ± 3.09E-07 | 1.43E-03 ± 1.03E-04 | 2 | 0.99724 |
| **243** | 1.3183 ± 0.0131 | 1.17E-07 ± 2.31E-09 | 0.484 ± 0.002 | 2.49E-07 ± 5.17E-09 | 86 | 3.09E-04 ± 2.08E-06 | 1.41E-04 ± 9.43E-07 | 12 | 5.05E-06 ± 2.98E-07 | 1.27E-03 ± 7.53E-05 | 2 | 0.99736 |
| **273** | 1.1665 ± 0.0105 | 1.57E-07 ± 2.75E-09 | 0.522 ± 0.002 | 2.91E-07 ± 5.33E-09 | 87 | 3.01E-04 ± 1.96E-06 | 1.43E-04 ± 9.05E-07 | 11 | 3.91E-06 ± 2.37E-07 | 1.76E-03 ± 1.47E-04 | 2 | 0.99738 |
| **293** | 1.0452 ± 0.0088 | 1.88E-07 ± 2.96E-09 | 0.556 ± 0.002 | 3.14E-07 ± 5.19E-09 | 88 | 2.57E-04 ± 1.65E-06 | 1.44E-04 ± 9.06E-07 | 10 | 3.30E-06 ± 1.93E-07 | 2.30E-03 ± 2.44E-04 | 2 | 0.99744 |
| **323** | 1.0032 ± 0.0074 | 2.02E-07 ± 2.68E-09 | 0.599 ± 0.002 | 3.04E-07 ± 4.23E-09 | 87 | 2.41E-04 ± 1.33E-06 | 1.49E-04 ± 8.01E-07 | 11 | 2.41E-06 ± 1.44E-07 | 2.95E-03 ± 4.02E-04 | 2 | 0.99787 |
| **353** | 0.9304 ± 0.0070 | 1.89E-07 ± 2.41E-09 | 0.644 ± 0.003 | 2.61E-07 ± 3.51E-09 | 83 | 2.71E-04 ± 1.37E-06 | 1.52E-04 ± 7.88E-07 | 15 | 2.53E-06 ± 1.79E-07 | 1.70E-03 ± 1.54E-04 | 3 | 0.99777 |
| **383** | 0.9701 ± 0.0085 | 1.30E-07 ± 1.84E-09 | 0.629 ± 0.003 | 1.85E-07 ± 2.74E-09 | 89 | 1.33E-04 ± 7.15E-07 | 1.47E-04 ± 8.76E-07 | 10 | 1.87E-06 ± 1.27E-07 | 1.14E-03 ± 6.76E-05 | 1 | 0.99766 |
| **413** | 1.0048 ± 0.0103 | 8.74E-08 ± 1.40E-09 | 0.629 ± 0.003 | 1.24E-07 ± 2.08E-09 | 92 | 1.32E-04 ± 7.97E-07 | 7.05E-05 ± 4.49E-07 | 7 | 2.03E-06 ± 1.43E-07 | 4.34E-04 ± 2.04E-05 | 1 | 0.9973 |
| **443** | 1.1003 ± 0.0218 | 5.47E-08 ± 1.63E-09 | 0.604 ± 0.005 | 8.16E-08 ± 2.54E-09 | 96 | 1.16E-04 ± 1.16E-06 | 3.06E-05 ± 1.32E-07 | 4 | | | | 0.99124 |
| **473** | 1.1470 ± 0.0237 | 3.84E-08 ± 1.14E-09 | 0.612 ± 0.005 | 5.63E-08 ± 1.74E-09 | 94 | 1.37E-04 ± 1.16E-06 | 2.56E-05 ± 8.14E-08 | 6 | | | | 0.99275 |
| **503** | 1.3674 ± 0.0333 | 2.26E-08 ± 7.77E-10 | 0.574 ± 0.005 | 3.61E-08 ± 1.29E-09 | 94 | 1.16E-04 ± 9.21E-07 | 2.42E-05 ± 6.98E-08 | 6 | | | | 0.99328 |

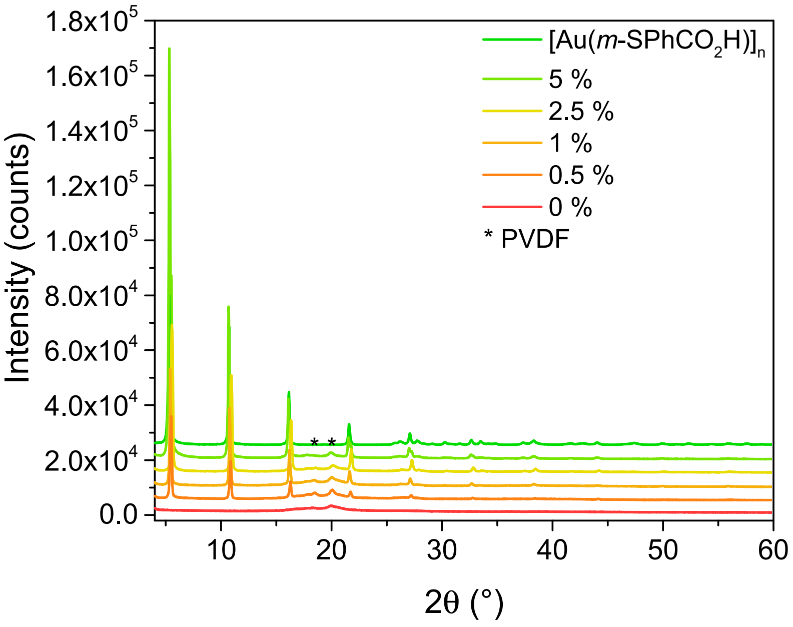


**Figure 11.** PXRD patterns of x %[Au(*m*-SPhCO$_2$H)]$_n$@PVDF films.

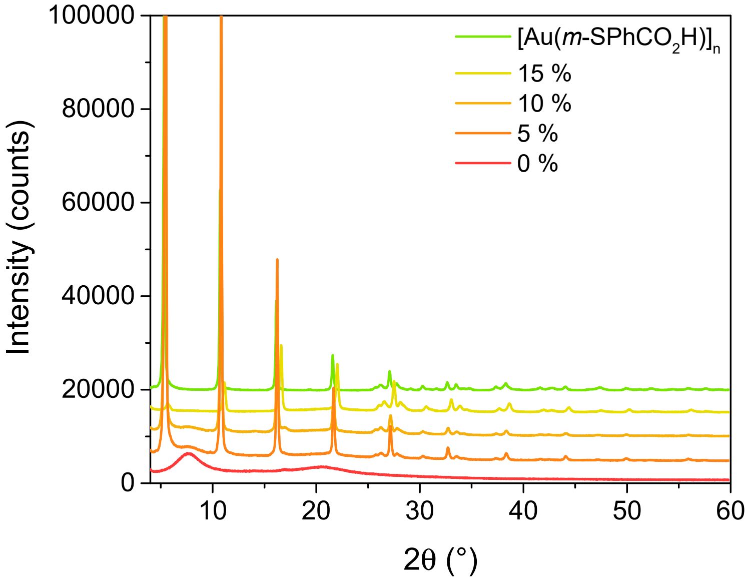


**Figure 12.** PXRD patterns of x %[Au(*m*-SPhCO$_2$H)]$_n$@PVK films.

## References


1. Veselska, O.; Okhrimenko, L.; Guillou, N.; Podbevšek, D.; Ledoux, G.; Dujardin, C.; Monge, M.; Chevrier, D. M.; Yang, R.; Zhang, P.; Fateeva, A.; Demessence, A., An intrinsic dual-emitting gold thiolate coordination polymer, [Au(+I)(*p*-SPhCO$_2$H)]$_n$, for ratiometric temperature sensing. *J. Mater. Chem. C* **2017,** *5*, 9843.
2. Lavenn, C.; Guillou, N.; Monge, M.; Podbevšek, D.; Ledoux, G.; Fateeva, A.; Demessence, A., Shedding light on an ultra-bright photoluminescent lamellar gold thiolate coordination polymer [Au(*p*-SPhCO$_2$Me)]$_n$. *Chem. Commun.* **2016,** *52*, 9063.
